# AVDS should not dethrone ARDS

**DOI:** 10.1186/s13054-021-03807-y

**Published:** 2021-11-18

**Authors:** Yazine Mahjoub, Daniel Rodenstein, Vincent Jounieaux

**Affiliations:** 1 Cardiac Vascular Thoracic and Respiratory Intensive Care Unit, Department of Anaesthesia and Intensive Care, Amiens University Medical Centre, Amiens, France; 2 Pneumology Department, Cliniques Universitaires Saint-Luc, Université Catholique de Louvain, Brussels, Belgium; 3 Pneumology Department, Amiens University Medical Centre, Amiens, France

**To the Editor**

We read with great interest the commentary by Gattinoni and Marini [1] trying to “*accommodate the discordant and inconvenient Covid-19 observations*.” In their analysis, the authors suggest to modify, rearrange, rethink the Adult Respiratory Distress Syndrome (ARDS) and even abandon it because in the early stage of COVID-19, lungs are unusually compliant and gas filled end thus differ from ARDS related to other diseases. We agree that COVID-19 atypical early presentation required a specific and unusual treatment. However, this does not imply that we should abandon the term ARDS and all physiopathology and logic that lies behind it. This acronym has helped to promote lung protective strategy, Peep trial, prone positioning and recruitment maneuvers that have saved many lives, or at least have avoided adding iatrogenic injury to disease-related lung insults. At the start of the first wave of the COVID-19 pandemic, we proposed to consider COVID-19 as, indeed, a new type of disease that we termed AVDS (Acute Vascular Distress Syndrome), that affects vessels, mainly pulmonary vessels, leading to an intrapulmonary shunt [2]. COVID-19 is histologically characterized with lung endothelium damage and significant neo-angiogenesis. This pattern has also been characterized on thoracic imaging (pulmonary arterial dilatations on CT-scan, areas with increased pulmonary flow on dual energy CT-scan or on late images from ventilation-perfusion pulmonary scintigraphy [3]). This early vascular insult explains the so-called mysterious happy hypoxia [4], ICU presentation with increased compliance, low recruitability [5], increased cardiac output with low vascular pulmonary resistances and increased intrapulmonary shunt. Some other ICU data have support the AVDS concept showing relative inefficacy of arterial vasodilatator agent such as iNO and the benefit of the arterial vasoconstrictor agent almitrine. All these observations led us to propose the acronym AVDS to describe the early stage of the COVID-19 disease [2]. With the progression of the disease appeared the alveolar insult (which may be more the consequence of the endothelium injury than a direct alveolar injury) that hides the persisting vascular insult. At this time, if the alveolar injury overcomes the vascular one, the presentation may be a typical ARDS and patients may still require the classic ARDS treatments including the ARDS recommended lung protective strategy.

Therefore, we consider it more useful and truthfully to add AVDS to ARDS rather than to abandon a concept that has proved useful and still keeps all its sense.

## Data Availability

Not applicable.

## References

[1] Gattinoni L, Marini JJ (2021). Isn't it time to abandon ARDS? The COVID-19 lesson. Crit Care.

[2] Mahjoub Y, Rodenstein DO, Jounieaux V (2020). Severe Covid-19 disease: rather AVDS than ARDS?. Crit Care.

[3] Jounieaux V, Mahjoub Y, El-Esper I, Rodenstein DO (2021). The importance of lung hyperperfusion patterns in COVID-19-related AVDS. Eur J Nucl Med Mol Imaging.

[4] Jounieaux V, Rodenstein DO, Mahjoub Y (2020). On happy hypoxia and on sadly ignored "acute vascular distress syndrome" in patients with COVID-19. Am J Respir Crit Care Med.

[5] Abou-Arab O, Haye G, Beyls C, Huette P, Roger PA, Guilbart M, Bernasinski M, Besserve P, Trojette F, Dupont H, Jounieaux V, Mahjoub Y (2021). Hypoxemia and prone position in mechanically ventilated COVID-19 patients: a prospective cohort study. Can J Anaesth.

