# Supplementary material for: AVDS should not dethrone ARDS
Source: Crit Care. 2021 Nov 18;25:400. doi: 10.1186/s13054-021-03807-y (PMC8600342; doi:10.1186/s13054-021-03807-y)
Supplement: Related texts

# Reply: Authors’ response

**Authors:** J. J. Marini, L. Gattinoni

**Affiliations:** 4 grid.17635.36 0000000419368657 Department of Pulmonary and Critical Care Medicine, University of Minnesota and Regions Hospital, St. Paul Minnesota, USA; 5 grid.411984.1 0000 0001 0482 5331 Department of Anesthesiology, University Medical Center Göttingen, Robert-Koch Straße 40, 37075 Göttingen, Germany

**To the Editor,**

We appreciate the thoughtful letter of Mahjoub and colleagues, whose perspective, while somewhat differently expressed, is quite compatible with ours. Our correspondents apparently read the title of our commentary but seem not to have digested the spirit of our text. Certainly, we did not intend in our choice of a deliberately provocative title to encourage an abandonment ‘all the physiology and logic that lies behind’ the label of ARDS. Indeed, we have devoted much of our investigative careers to defining more clearly the drivers of acute lung injury and its safe management. We seem to both agree that the label of ARDS is a very broad problem category that requires more than one treatment approach, depending on its specific cause. COVID pneumonia has provided a good example of what can go wrong with a standardized ‘lung protective’ prescription, as both of our groups have argued in the literature. (We also agree with Majhoub and colleagues that the term AVDS may indeed a step toward directing our clinical focus on the actual respiratory disturbances of this acute, and progressive lung affliction—and to avoid the errors of using ‘standardized’ dictates for ARDS ventilation therapy.) We might disagree, however, that the acronym ARDS *itself* has ‘helped promote lung protective strategy, PEEP strategy, prone positioning, recruitment maneuvers that have saved many lives.’ Deeper understanding of the varied forms and properties of acute lung injury, patient ventilator interactions and the hazards of VILI did. Indeed, the lumping of patients into that unifying label in RCTs has contributed to the obfuscation of the nuances of appropriate treatment. Sounding the ‘fire alarm’ bell of ARDS does not tell you what is causing the conflagration nor how best to address the hazard. In our opinion, ARDS should be viewed much like the terms chronic heart failure (CHF) and renal failure. CHF does not have a single approach, but one based on personalized data collection, a pathophysiologic understanding of what is causing the disturbance, and a logical approach to its therapy. We thank Mahjoub and colleagues for reinforcing the central gist of our intended message.
